# Incidental findings on cardiac computed tomography in incident hemodialysis patients: the predictors of arrhythmic and cardiovascular events in end-stage renal disease (PACE) study

**DOI:** 10.1186/1471-2369-15-68

**Published:** 2014-05-01

**Authors:** Bernard G Jaar, Lili Zhang, Svetlana V Chembrovich, Stephen M Sozio, Tariq Shafi, Julia J Scialla, Gordon F Tomaselli, Joao A C Lima, Wen Hong Linda Kao, Rulan S Parekh, Lucy A Meoni

**Affiliations:** 1 Department of Medicine, Johns Hopkins School of Medicine, Baltimore, MD, USA; 2 Department of Epidemiology, Johns Hopkins Bloomberg School of Public Health, Baltimore, MD, USA; 3 Welch Center for Prevention, Epidemiology and Clinical Research, Johns Hopkins Medical Institutions, Baltimore, MD, USA; 4 Nephrology Center of Maryland, Baltimore, MD, USA; 5 Jacobi Medical Center, Bronx, NY, USA; 6 Greater Baltimore Medical Center, Baltimore, MD, USA; 7 Department of Medicine, University of Miami Miller School of Medicine, Miami, FL, USA; 8 Departments of Pediatrics, Hospital for Sick Children and Medicine, University Health Network, University of Toronto, Ontario, Canada; 9 Department of Biostatistics, Johns Hopkins Bloomberg School of Public Health, Baltimore, MD, USA

**Keywords:** Incidental findings, Cardiac, Computed tomography, Hemodialysis, Prevalence, Pulmonary nodule

## Abstract

**Background:**

This is the first study that has examined non-cardiac incidental findings in research cardiac computed tomography (CT) of hemodialysis patients and their relationship with patient characteristics.

**Methods:**

We performed a cross-sectional analysis in the Predictors of Arrhythmic and Cardiovascular Events in End-Stage Renal Disease (PACE) study, a prospective cohort study on incident hemodialysis patients. Non-cardiac structures in the cardiac CT scan were reviewed and evaluated. The type and frequencies of non-cardiac incidental CT findings were summarized. Univariate and multivariate logistic regression were performed to analyze the associations between gender, older age, obesity, history of cardiovascular disease (CVD), smoking status, history of chronic pulmonary disease and history of cancer with presence of any incidental CT findings and, separately, pulmonary nodules.

**Results:**

Among the 260 participants, a total of 229 non-cardiac incidental findings were observed in 145 participants (55.8% of all participants). Of these findings, pulmonary nodules were the most common incidental finding (24.2% of all findings), and 41.3% of them requiring further follow-up imaging per radiology recommendation. Vascular and gastrointestinal findings occurred in 11.8% and 15.3% of participants, respectively. Participants 65 years or older had a higher odds of any incidental findings (Odds Ratio (OR) =2.55; 95% Confidence Intervals (CI) 1.30, 4.99) and pulmonary nodules (OR = 4.80; 95% CI 2.51, 9.18). Prior history of CVD was independently and significantly associated with any incidental findings (OR = 2.00; 95% CI 1.19, 3.40); but not with the presence of pulmonary nodules.

**Conclusions:**

We demonstrate that the prevalence of incidental findings by cardiac CT scanning is extremely high among patients on hemodialysis. Further investigations to follow-up on the high occurrence of incidental findings during our research study and potentially clinical studies raises important practical, ethical and medico-legal issues that need to be carefully considered in research projects using imaging studies.

## Background

Cardiac computed tomographic (CT) angiography is a powerful noninvasive technique for the evaluation of coronary artery disease (CAD) and cardiac structure. An important advantage of cardiac CT over other noninvasive tests for CAD is its ability to directly visualize coronary arteries, aortic, and other cardiac structures and determine additional measures of calcification of coronaries and valves. Simultaneously, portions of non-cardiac structures in the chest and upper abdomen are also visible on the scan and abnormalities in these anatomic regions can be detected. There have been several studies among non-dialysis patients with suspected CAD describing non-cardiac incidental CT findings [1-12]. The prevalence of these non-cardiac incidental findings ranges from 8% to 69% depending on the type of CT protocol used and age of the participants; with the most frequent incidental CT finding being pulmonary nodules. There are few studies investigating incidental CT findings in chronic conditions such as those patients on chronic dialysis where CT imaging for clinical indications is done frequently.

End-stage renal disease (ESRD) patients experience a very high incidence of cardiovascular events, particularly sudden cardiac death, possibly caused by left ventricular hypertrophy, electrolyte derangement and vascular calcification among other causes [13,14]. In the Predictors of Arrhythmic and Cardiovascular Events in End-Stage Renal Disease (PACE) study, incident hemodialysis patients underwent cardiac CT scan for the evaluation of coronary artery disease as well as coronary and valvular calcification. Meanwhile, non-cardiac structures in the CT scan were also reviewed and evaluated on a routine basis.

To our knowledge no other study has reported the prevalence of incidental findings in cardiac CT scans of hemodialysis patients and their relationship with patient characteristics. Therefore, the current study sought to describe the prevalence of non-cardiac incidental findings by cardiac CT in incident hemodialysis patients, to demonstrate the nature and frequency of different incidental findings in this population and to investigate the potential associations of hemodialysis patients’ characteristics with these incidental findings.

## Methods

### Study population

The Predictors of Arrhythmic and Cardiovascular Risk in ESRD (PACE) study is an ongoing prospective cohort study in the greater Baltimore area in Maryland, with the primary goal of investigating risk factors for sudden cardiac death and disorders of cardiac autonomic regulation and ventricular conduction in patients with incident ESRD treated with hemodialysis. Enrollment began in November 2008 and participants were recruited through identifying and screening of incident participants in the outpatient dialysis units (24 DaVita units and 2 MedStar Units). Eligible hemodialysis patients were aged 18 years or older, started hemodialysis within six months of enrollment and were able to provide informed consent. Participants were excluded if they were on home dialysis, had active cancer other than nonmelanoma skin cancer, had a pacemaker/automatic implantable cardioverter defibrillator, or were pregnant or nursing mothers. At baseline, information on participants’ demographics, medical history, questionnaires (including physical activity, cognitive function, quality of life, frailty and diet), imaging studies (including cardiac CT and echocardiogram), pulse wave velocity, ankle-brachial index, electrocardiograms and blood samples were collected on nondialysis days. Participants were followed every 6 months by telephone for medical history and diet and every 12 months with repeated electrocardiograms, pulse wave velocity, ankle-brachial index, blood collection and follow-up questionnaires. Participants’ medical records were thoroughly reviewed by trained physician adjudicators to assess baseline comorbidities at initiation of hemodialysis.

The study protocol was approved by the institutional review board of The Johns Hopkins School of Medicine, MedStar Health Systems and by the medical director of each dialysis unit. All participants provided informed written consent. The current cross-sectional analysis included the first 260 participants who underwent a cardiac CT measurement as part of the PACE study protocol and had data collected during the enrollment study visit.

### Assessment of outcomes

Each participant underwent a cardiac CT using the Toshiba Aquilon One, 320-row detector CT scanner (Tokyo, Japan) as part of the PACE protocol for quantification of the burden of coronary atherosclerosis and calcification. The study protocol minimized radiation to 5–7 Millisievert (mSv). Minimal dose of non-ionic, low osmolar contrast was also given to the participants without a contraindication to intravenous contrast. A single prospective ECG-triggered acquisition was acquired in mid-diastole within one R to R interval of a single heartbeat. After the completion of the scan, a trained cardiologist reviewed the CT images to determine if there was a critical/urgent alert based on clinical judgment. At the request of the investigators, trained radiologists subsequently read independently all CT images to assess for non-cardiac pathologies and submitted a separate report of these findings within a week. To handle critical or urgent alerts, the cardiologist and radiologist were provided with a weekly call schedule including the PACE investigators (BGJ, SMS, TS, JJS) contact information. Further, the PACE investigators had full access to a database containing the participants contact information, emergency contact person and their physicians’ name and contact information.

### Assessment of other variables

Age, sex, and race were self-reported. Body mass index (BMI) was calculated as weight in kilograms divided by the square of the height in meters. “Ever smoking” was defined as self-reported lifetime exposure of at least 100 cigarettes (approximately 5 packs). Existing cardiovascular disease (CVD) was defined as self-reported or physician-diagnosed history of coronary artery disease, prior coronary revascularization by balloon angioplasty, stenting or bypass surgery, heart failure, stroke, transient ischemic attack or peripheral vascular disease. Prevalent chronic lung disease was defined as self-reported, physician diagnosed asthma or reactive airway disease, or ever being treated for these conditions. History of cancer was self-reported if diagnosed or treated within the last 5 years of starting dialysis.

### Statistical analysis

We performed Pearson’s chi-squared test to examine if the distribution of categorical variables (male gender, white race, age ≥ 65 years, BMI ≥ 30 kg/m^2^, history of CVD, smoking status, history of chronic lung disease, history of cancer) were similar between participants with and without incidental findings and between participants with and without pulmonary nodules, the most common finding in our population. Logistic regression was performed to analyze the univariate associations between male, older age, obesity, smoking status, history of CVD, chronic lung disease and cancer with the presence of any incidental CT findings and/or pulmonary nodules. Multivariate logistic regression adjusted for age, gender, BMI, smoking status and history of CVD was performed to account for potential confounders. Associations were presented as odds ratios (OR), 95% confidence intervals and p values less than 0.05 were considered statistically significant. Stata 11 (StataCorp LP, College Station, Texas, USA) was used for analysis.

## Results

Among the first 260 participants who completed the cardiac CT scanning protocol, the mean age of the participants was 55 years (standard deviation (SD) ± 13.5 years) and 57.7% of them were male. A total of 229 non-cardiac incidental findings were observed in 145 participants (55.8% of all participants), and 42 participants (16.2% of all participants) had two or more non-cardiac incidental findings. Of these findings, pulmonary nodules were the most common incidental finding observed in 63 participants (24.2% of all participants), and 41.3% of them required further follow-up imaging per radiology recommendation.

**Figure 1:**
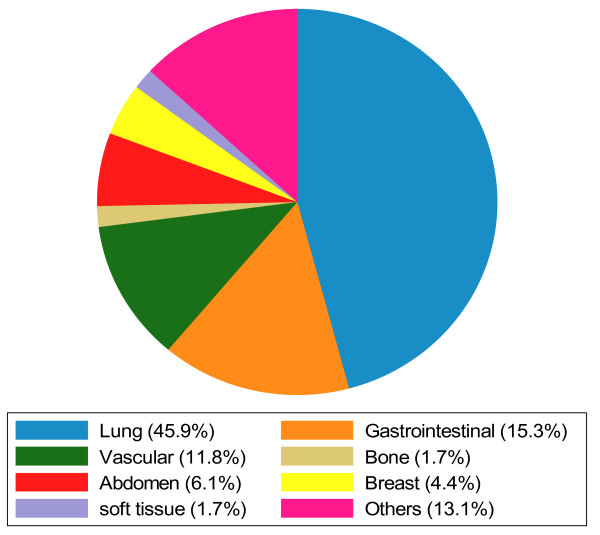
Distribution of non-cardiac incidental CT findings by organ system among 260 incident hemodialysis study participants.

**Figure 2:**
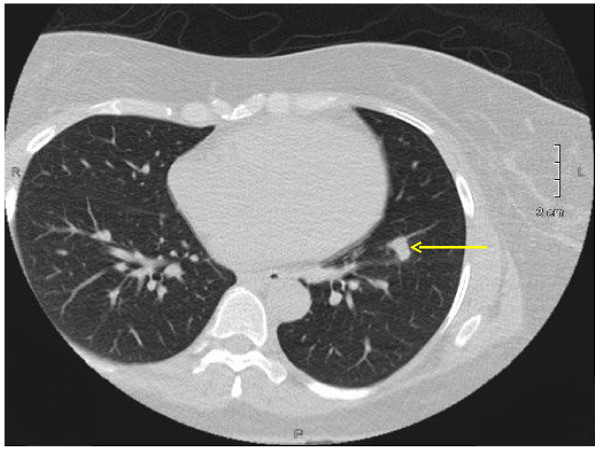
A 1.3 cm × 0.8 cm nodule in the left lung lower lobe.

**Figure 3:**
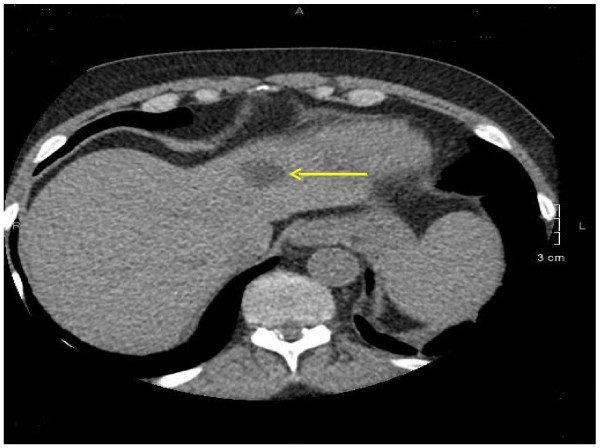
A left liver lobe lobulated hypodense lesion 2.2 × 1.9 cm.

The nature and frequency of the clinically significant incidental findings are presented in Table 1. Besides pulmonary nodules (n = 63), the five most common non-cardiac incidental findings were pleural effusions (n = 23), pericardial effusions (n = 23), hiatal hernias (n = 17), mediastinal lymph nodes (n = 16) and aortic aneurysms (n = 14); two associated with dissection. Of the 63 nodules, 46 (73%) of them were less than 6 mm; 11 (17.5%) of them were between 6 and 9 mm and 6 (9.5%) of them were more than 10 mm. The distribution of regions of the 229 non-cardiac incidental findings are presented in Figure 1. Nearly half (45.9%) of the incidental findings were observed in the pulmonary region. Figure 2 illustrates a pulmonary nodule. The gastrointestinal (15.3%) and vascular regions (11.8%) also had high prevalence of abnormalities. Other findings were observed in the abdomen (Figure 3 illustrates a liver lesion), but also breast, bone and soft tissue regions in a descending order. Most notably, we discovered 5 participants with pulmonary embolism who were immediately referred to the emergency department for further evaluation and treatment.

**Table 1:** Type and frequency of non-cardiac incidental CT findings among 260 incident hemodialysis study participants

**Incidental findings**	**Number (%)**
Pulmonary nodule*	63 (27.5)
Pleural effusion	23 (10.0)
Pericardial effusion	23 (10.0)
Hiatal hernia	17 (7.4)
Mediastinal lymph node**	16 (7.0)
Aortic aneurysm	14 (6.1)
Pulmonary cyst bleb	9 (4.0)
Pulmonary embolism	5 (2.2)
Abdominal mass***	5 (2.2)
Thrombus of central venous catheter	3 (1.3)
Other	51 (22.3)
**Total**	**229**

*Nodules: 46 (73%) of them less than 6 mm; 11 (17.5%) of them between 6 and 9 mm and 6 (9.5%) of them more than 10 mm.

**Mediastinal lymph nodes: 10 (62.5%) of them less than 10 mm and 6 (37.5%) of them 10 mm or more.

***Abdominal mass: 4 of them were in the liver and one was in the left adrenal gland.

The characteristics of the participants by their status of any incidental findings and pulmonary nodules are presented in Table 2. The percentage of older participants (age ≥ 65 years) was much greater in the group with any incidental findings (29.7% versus 13%) and with pulmonary nodules (46% versus 14.7%) than their counterparts (P = 0.001 and P < 0.001, respectively). Participants with no incidental findings were more likely to be obese (BMI ≥ 30 kg/m^2^) than participants with any incidental findings (P = 0.009); however, this association was not significant when comparing participants with and without pulmonary nodules (P = 0.6). The percentage of participants with prior CVD was significantly higher in the group with any incidental findings (53.5% versus 33.9%) and with pulmonary nodules (55.6% versus 41.1%) than their counterparts (p = 0.002 and p = 0.048, respectively). Interestingly, there was no difference between the groups by gender, race, smoking status, history of chronic lung disease and history of cancer (P > 0.05).

**Table 2:** Characteristics of 260 hemodialysis study participants by incidental findings and pulmonary nodules

	**No incidental findings**	**Any incidental findings**		**No pulmonary nodules**	**Presence of pulmonary nodules**	
	**n = 115**	**n = 145**		**n = 197**	**n = 63**	
	**n (%)**	**n (%)**	**P value**	**n (%)**	**n (%)**	**P value**
Male	63 (54.8)	87 (60.0)	0.4	82 (41.6)	28 (44.4)	0.7
Whites	30 (26.1)	44 (30.3)	0.5	50 (25.4)	24 (38.1)	0.05
Age ≥ 65 years	15 (13.0)	43 (29.7)	0.001	29 (14.7)	29 (46.0)	<0.001
BMI* ≥ 30 kg/m^2^	49 (42.6)	39 (27.1)	0.009	65 (33.0)	23 (36.5)	0.6
Prevalent CVD	39 (33.9)	77 (53.5)	0.002	81 (41.1)	35 (55.6)	0.048
Ever smoking	64 (55.7)	89 (61.8)	0.3	111 (56.4)	42 (66.7)	0.2
Chronic pulmonary disease	26 (22.8)	27 (19.0)	0.5	37 (18.8)	16 (25.4)	0.3
Cancer	6 (5.3)	6 (4.2)	0.678	10 (5.1)	2 (3.2)	0.522

*BMI: body mass index.

Clinical correlates including male gender, older age, obesity, smoking status, history of CVD, chronic lung disease and cancer associated with the presence of any incidental findings or pulmonary nodules are presented in Table 3. Incident hemodialysis participants who were 65 years or older had approximately three times higher odds of having any incidental CT findings (OR = 2.81, 95% CI 1.47, 5.38) and five times higher odds of having pulmonary nodules (OR = 4.94, 95% CI 2.62-9.31) compared to participants less than 65 years old. Similarly, participants with a history of CVD had a significantly higher odds of any incidental CT findings (OR = 2.24, 95% CI 1.35, 3.72) and pulmonary nodules (OR = 1.77, 95% CI 1.00, 3.15) compared with participants without prior CVD. On the other hand, obese participants had a significantly lower odds of having any incidental CT findings (OR = 0.5, 95% CI 0.30, 0.84) compared to non-obese participants. There was no association between obesity and finding of pulmonary nodules (OR = 1.16, 95% CI 0.64, 2.10).

**Table 3:** Association with the presence of any incidental CT findings and pulmonary nodules among 260 incident hemodialysis study participants

		**Univariate model**		Multivariate model*	
**Outcomes**	**Variables**	**Odds ratio (95% CI)**	**P value**	**Odds ratio (95% CI)**	**P value**
Any incidental CT findings	Male	1.24 (0.56, 2.03)	0.40	1.12 (0.65, 1.90)	0.71
	Age ≥ 65 years	2.81 (1.47, 5.38)	**0.002**	2.55 (1.30, 4.99)	**0.006**
	BMI** ≥ 30 kg/m^2^	0.50 (0.30, 0.84)	**0.009**	0.53 (0.30, 0.92)	**0.024**
	Prevalent CVD***	2.24 (1.35, 3.72)	**0.002**	2.00 (1.19, 3.40)	**0.009**
	Ever smoking	1.29 (0.78, 2.12)	0.32	1.09 (0.64, 1.85)	0.76
	Chronic pulmonary disease	0.80 (0.43, 1.46)	0.46	0.87 (0.44, 1.72)	0.69
	History of cancer	0.78 (0.24, 2.50)	0.68	0.39 (0.10, 1.47)	0.17
Pulmonary Nodule	Male	0.89 (0.50, 1.58)	0.69	0.96 (0.50, 1.82)	0.89
	Age ≥ 65 years	4.94 (2.62, 9.31)	**<0.001**	4.80 (2.51, 9.18)	**<0.001**
	BMI** ≥ 30 kg/m^2^	1.16 (0.64, 2.10)	0.63	1.28 (0.65, 2.48)	0.48
	Prevalent CVD***	1.77 (1.00, 3.15)	**0.05**	1.42 (0.77, 2.62)	0.26
	Ever smoking	1.53 (0.85, 2.78)	0.16	1.50 (0.79, 2.86)	0.22
	Chronic pulmonary disease	1.48 (0.75, 2.89)	0.26	1.55 (0.73, 3.29)	0.26
	History of cancer	0.61 (0.13, 2.85)	0.53	0.21 (0.04, 1.06)	0.06

*Multivariate model adjusted for gender, age, BMI, smoking status, chronic pulmonary disease, history of cancer and prevalent CVD.

**BMI: body mass index.

***CVD: cardiovascular disease.

In the multivariate model (Table 3), after adjusting for age, gender, BMI, smoking status and history of CVD, these associations were slightly attenuated, but remained statistically significant, except for the association between prior history of CVD and pulmonary nodules. Male gender, smoking status, history of chronic lung disease and cancer were not associated with the presence of any incidental findings or pulmonary nodules. In sensitivity analyses, after removing pulmonary nodules from the incidental findings, obese participants were still significantly less likely to have any incidental findings compared to non-obese participants. Further, adding race to the multivariate model did not modify the observed associations.

## Discussion

In this cross-sectional study, we demonstrate that the prevalence of incidental findings by cardiac CT scanning is extremely high among persons starting hemodialysis therapy undergoing a study for research purposes and not clinical indications. Pulmonary nodules were the most frequently encountered incidental finding. Also, among participants age 65 and older, the mean age of the US dialysis population, there was a three times higher odds of non-cardiac incidental findings and they were almost five times more likely to have pulmonary nodules discovered. Prior history of CVD was independently and significantly associated with any incidental findings, but not with the presence of pulmonary nodules. In contrast, obese participants did not have a higher presence of any incidental findings. Interestingly, neither smoking status nor history of chronic pulmonary disease was associated with any incidental findings or pulmonary nodules. The cardiovascular imaging studies were conducted in the context of the high burden of cardiovascular disease among patients on dialysis; however, they were collected as part of a clinical research study in asymptomatic conditions. The findings of this study highlight some important issues related to conducting imaging studies for dialysis patients often required for clinical evaluation in practice. Often incidental findings require follow-up and potentially further imaging using ionizing radiation to reassess the initial findings, follow-up for progression and potential intervention.

To the best of our knowledge, the current study is the first to describe the non-cardiac incidental CT findings among patients recently initiated on maintenance hemodialysis. These findings expand our current knowledge. First, a clear description of the nature and frequency of the non-cardiac incidental findings enables a better understanding of the pathologic spectrum of the chest and upper abdomen regions detected by cardiac CT screening in this high-risk population. The high prevalence of incidental CT findings and pulmonary nodules in our population is similar or higher than what has been reported in previous studies depending on the patient populations [6,9,11,14,15]. Second, our study implemented a CT reading protocol including timely preliminary reading and careful final reading, which not only makes sure that we identified any critical/urgent alerts quickly, but also enables all CT images being systematically reviewed without compromising validity. Importantly, this protocol potentially saved the life of some asymptomatic participants with pulmonary embolism and even thoracic aortic aneurysm. The study investigators and the participants’ physicians were immediately notified and necessary management steps were taken avoiding potentially life-threatening consequences. In these cases, our participants immediately and directly benefited from the research cardiac CT scan. Despite the significant benefit seen in these study participants, there are a greater number of participants that will require follow-up based on the pulmonary nodules with additional ionizing radiation and incurring more costs to the medical system. The risks and benefits to study participants need to be considered in order to determine the incremental net benefit of the research study aims versus the risk of incidental findings.

In the current analysis, we examined associations between common sociodemographic and clinical risk factors likely to be associated with incidental findings and pulmonary nodules. Not too surprisingly, older age was significantly associated with both incidental findings and pulmonary nodules. Obesity and history of CVD were only significantly associated with any incidental findings, but not pulmonary nodules; however, this may be related to a smaller sample size or the association was driven mainly by other abnormalities. Interestingly, we found no association between smoking status or history of chronic pulmonary disease with either any incidental findings or pulmonary nodules. This again may be due to our limited sample size or maybe due to misclassification bias introduced by self-reported smoking history.

The results of the current study should be interpreted with some considerations in mind. First, there have been numerous publications describing the prevalence of incidental CT findings in different populations [1-12,16,17] ranging from 8% to 69%; however, no studies in the dialysis population have been published to our knowledge. These results should be considered and utilized with caution, as clinical imaging used in research requires necessary protocols to deal with the high volume of unexpected findings especially using new CT acquisition protocols, better equipment resulting in improved anatomic definition and increased chances of more incidental findings. Generally, cardiac CT scan is viewed separately from pulmonary scans due to the difference in scanning region and CT modalities [15] and non-contrast calcium scoring CT has lower power in detecting incidental findings than coronary CT angiography [14]. The researchers’ obligation of identifying incidental findings and informing research participants remains a controversial issue as the clinical follow-up may require significant and immediate intervention. Additionally, there needs to be an organized plan for a timely reading of the CT scan with eventual clinical follow-up but also importantly the debate focuses on the costs of causing increased anxiety and unnecessary exposure to further radiation. The long-term treatment and the benefits of preventing diseases for incidental findings are not clear. The most recent recommendations classified incidental findings into three categories: “strong net benefit”; “possible net benefit”; and “unlikely net benefit”, depending on the severity of the incidental findings and the possibility of avoiding and ameliorating these conditions and the recommended action for these three categories in terms of disclosure to research participants are “disclose”, “may disclose” and “do not disclose”, respectively [18]. Large-field reconstruction for pulmonary cancer detection and serial CT scans to follow-up pulmonary nodules are not recommended due to high false positive rate and limited benefits [19]. A well-accepted guideline was proposed by the Fleischner Society in 2006 [20]. The recommended follow-up varies depending on the size of the pulmonary nodules and patients’ risk factors. In research studies, however, it may be difficult to evaluate study participants’ risk if such information is not within the scope of the study protocol.

Nevertheless, some limitations of the current study need to be considered. Since the primary aim of performing cardiac CT scan is to measure coronary calcium score and atherosclerosis, there may be some self-selection with sicker patients enrolling in the study because of free access to several cardiovascular tests. For those suspicious nodules undergoing further evaluation, we were not able to acquire follow-up information about the final clinical outcome since this was outside the scope of the study and with limited follow-up time. As a result, the validity and false positive rate of these incidental findings could not be assessed. Finally, we found no association between pulmonary nodules and smoking status, history of chronic lung disease or cancer. The smaller sample size of our cohort may have led to this lack of statistical association. However, other important potential explanations for this lack of statistical association could be due to the exclusion of patients with active cancer from the cohort and also to the relatively younger age of our population (mean age of 55 years). Because of the characteristics of our cohort, these results may not be generalizable to the entire hemodialysis population in the United States.

## Conclusions

Incidental findings by cardiac CT scan are extremely common among dialysis patients and pulmonary nodules are the most common findings. The current study, for the first time, describes the prevalence, the nature and frequency of non-cardiac CT findings captured by cardiac CT screening in asymptomatic incident hemodialysis participants. The excessive occurrence of these incidental findings requiring further investigations in this high-risk population raises important practical, ethical and medico-legal issues that need to be carefully considered in research projects using imaging studies.

## Abbreviations

CT: Computed tomography; CVD: Cardiovascular disease; OR: Odds Ratio; CI: Confidence Intervals; CAD: Coronary artery disease; ESRD: End-stage renal disease; mSv: Millisievert; BMI: Body mass index; SD: Standard deviation.

## Competing interests

The authors declare that they have no competing interest.

## Authors’ contributions

BGJ participated in study conception and design, analysis planning, determination of inferences, and drafting of the manuscript. LZ conducted the statistical analysis and participated in analysis planning, determination of inferences and drafting of the manuscript. SVC participated in analysis planning, chart review, determination of inferences and critical review of the manuscript. SMS participated in analysis planning, chart review, determination of inferences and critical review of the manuscript. TS participated in analysis planning, determination of inferences and critical review of the manuscript. JJS participated in analysis planning, chart review, determination of inferences and critical review of the manuscript. GFT participated in study conception and design, analysis planning, determination of inferences, and critical review of the manuscript. JACL participated in study conception and design, analysis planning, determination of inferences, and critical review of the manuscript. WHLK participated in study conception and design, analysis planning, determination of inferences, and critical review of the manuscript. RSP participated in study conception and design, analysis planning, determination of inferences, and critical review of the manuscript. LAM participated in study conception and design, analysis planning, statistical analysis, determination of inferences, and critical review of the manuscript. All authors read and approved the final manuscript.

## Pre-publication history

The pre-publication history for this paper can be accessed here:

http://www.biomedcentral.com/1471-2369/15/68/prepub
